# Prediction of late ventricular arrhythmias in patients with left ventricular assist device: insights from the VT-LVAD consortium

**DOI:** 10.1093/europace/euaf297

**Published:** 2025-11-17

**Authors:** Raphael Martins, Vincent Galand, Erwan Flecher, Pierre Groussin, Kerstin Bode, Elena Efimova, Alexey Dashkevich, Jackson Liang, John Larson, Blandine Mondesert, Jacinthe Boulet, Pierre-Emmanuel Noly, Frederic Sacher, Jean Luc Pasquié, Jean-Baptiste Gourraud, Sandro Ninni, Laurence Jesel, Alexandre Sebestyen, Vincent Algalarrondo, Jean-Claude Deharo, Frederic Anselme, Laure Champ-Rigot, Charles Guenancia, Bertrand Pierre, Romain Eschalier, Mathieu Echivard, Pierre Baudinaud, Nicolas Lellouche, Kevin Gardey, Karim Benali, Paul Gautier, Clément Delmas, Miloud Cherbi

**Affiliations:** Cardiology Department, University of Rennes, CHU Rennes, CIC 1414, INSERM, LTSI-UMR 1099, Rennes F-35000, France; Interventional Cardiology Department, Saint Joseph Clinic, Trélazé, France; Cardiology Department, University of Rennes, CHU Rennes, CIC 1414, INSERM, LTSI-UMR 1099, Rennes F-35000, France; Cardiology Department, University of Rennes, CHU Rennes, CIC 1414, INSERM, LTSI-UMR 1099, Rennes F-35000, France; Department of Electrophysiology, Heart Center Leipzig, Leipzig, Germany; Department of Electrophysiology, Heart Center Leipzig, Leipzig, Germany; Department of Cardiac Surgery, Heart Center Leipzig, Leipzig, Germany; Division of Cardiology, University of Michigan, Ann Arbor, MI, USA; Department of Cardiovascular Disease, Emory University, Atlanta, GA, USA; Montreal Heart Institute, Department of Medicine, Division of Cardiology, Université de Montréal, Montreal, QC, Canada; Montreal Heart Institute, Department of Medicine, Division of Cardiology, Université de Montréal, Montreal, QC, Canada; Montreal Heart Institute, Department of Medicine, Division of Cardiology, Université de Montréal, Montreal, QC, Canada; IHU LIRYC, CHU Bordeaux, Bordeaux University, INSERM 1045, Bordeaux F33000, France; Department of Cardiology, University Hospital of Montpellier, Montpellier, France; Department of Cardiology, CHU Nantes, Nantes, France; University of Lille, Inserm, CHU Lille, Institut Pasteur de Lille, Lille, France; Department of Cardiology, University Hospital of Strasbourg, Strasbourg, France; Department of Cardiac Surgery, University Hospital of Grenoble Alpes, Grenoble, France; Department of Cardiology, CHU Bichat, APHP, Paris, France; Assistance Publique-Hôpitaux de Marseille, Centre Hospitalier Universitaire La Timone, Service de Cardiologie, France and Aix Marseille Université, C2VN, Cardiologie-Hôpital La Timone, Boulevard Jean Moulin, Marseille 13005, France; Department of Cardiology, Rouen University Hospital and UNIROUEN, INSERM U1096, Rouen, France; Department of Cardiology, Caen University Hospital, Normandie University, UniCaen, Caen, France; Cardiology Department, University Hospital, Dijon, France; Department of Cardiology, University Hospital of Tours, Tours, France; Cardiology Department, CHU Clermont-Ferrand, Clermont-Ferrand, France; Department of Cardiology, CHRU Nancy, Vandoeuvre-les-Nancy, France; Division of Cardiology, European Georges Pompidou Hospital, Paris, France; Université Paris Cité, INSERM U970, Paris Cardiovascular Research Centre, Paris, France; Department of Cardiology, Hôpital Henri Mondor, Creteil, France; Cardiac Electrophysiology Department, Institut cardiologique de Lyon, Hôpital cardiologique Louis-Pradel, Hospices civils de Lyon, Université Claude-Bernard Lyon 1, Bron 69500, France; CHU Saint-Etienne, Université Jean Monnet, Mines Saint-Étienne, Inserm U1059, SAINBIOSE, Saint-Étienne, France; Cardiology Department, Toulouse University Hospital, Toulouse, France; Cardiology Department, Toulouse University Hospital, Toulouse, France; Department of Cardiology, AZ Sint-Jan Hospital, Ruddershove 10, Bruges 8000, Belgium

**Keywords:** Left ventricular assist device, Ventricular arrhythmia, Ventricular tachycardia, Ventricular fibrillation, Electrical storm, Heartmate, Heartware

Ventricular arrhythmias (VAs) are highly prevalent complications in left ventricular assist device (LVAD) recipients, with late VAs—occurring beyond 30 days post-implantation—affecting 20–50% of patients at 1 year.^1,2^ Unlike early VAs, which are often triggered by acute perioperative factors such as electrolyte disturbances or suction events, late VAs primarily arise from the underlying arrhythmogenic substrate of the cardiomyopathy.^1,3,4^ Despite their substantial clinical burden, data on late VA characteristics and risk prediction remain limited, and management strategies are poorly standardized. The VT-LVAD score^4,5^ was recently proposed as a simple tool to identify patients at risk of late VAs based on six readily available clinical variables: early VAs (≤30 days) after implantation, prior VA history, absence of angiotensin-converting enzyme inhibitor therapy, heart failure duration > 12 months, pre-implantation atrial fibrillation, and idiopathic dilated cardiomyopathy. However, this score has never been validated in large multicentre cohorts. Therefore, this study aimed to validate the performance of the VT-LVAD score in predicting late VA occurrence using data from an international, multicentre LVAD registry.

The VT-LVAD Consortium is an international, retrospective, multicentre study including LVAD patients implanted with durable continuous-flow devices between 2006 and 2019 across tertiary centres in France, Germany, the USA, and Canada. Patients were stratified into four risk groups according to their VT-LVAD score^4^: low (0–1 point), intermediate (2–4 points), high (5–6 points), and very high (7–10 points). The primary endpoint was the occurrence of late VAs (occurring more than 30 days after LVAD implantation) at 3-year follow-up, defined as sustained (>30 s) ventricular tachycardia or ventricular fibrillation, or shorter episodes if treated medically, by external electrical shock, or by appropriate implantable cardioverter-defibrillator (ICD) therapy. Kaplan–Meier survival analysis was performed to assess freedom from late VAs across risk groups, with hazard ratios (HRs) computed through multivariate Cox regression adjusted for age, gender, pre-LVAD ejection fraction, LVAD device type, LVAD strategy (bridge-to-transplantation vs. destination therapy), heart failure medications after implantation (beta-blockers, angiotensin receptor blockers, mineralocorticoid receptor antagonists), and presence of ICD prior to implantation. The relationship between the VT-LVAD score and late VA risk was further assessed using fractional polynomial analysis to evaluate linearity. All tests were two-tailed with *P* ≤ 0.05 accepted as statistically significant. Analyses were performed using R (v4.3.2). This study was approved by the institutional review board or ethics committee of each participating centre.

The analysis included 1146 LVAD patients with a median follow-up of 20.6 months. Based on the VT-LVAD score, 114 patients (9.9%) were classified as low risk, 425 (37.1%) as intermediate, 319 (27.8%) as high, and 288 (25.1%) as very high risk. The median age was 59.4 years and 84.6% were men. Patients in higher risk groups were older and had more comorbidities including diabetes and hypertension and lower ejection fraction. Overall, 311 patients (27.1%) experienced late VAs after a median time of 12.4 months, with event rates of 7.9% in the low-risk, 13.6% in intermediate, 34.5% in high, and 46.5% in very high-risk groups. Kaplan–Meier analysis demonstrated clear risk stratification across groups (*Figure 1A*), with adjusted HRs of 1.77 (0.88–3.57) for intermediate, 4.84 (2.45–9.55) for high, and 7.39 (3.76–14.51) for very high-risk groups compared with the low-risk reference (*P* for trend < 0.01). When analysed as a continuous variable, the VT-LVAD score demonstrated a strong linear relationship with late VA risk (C-index = 0.7, *P* for non-linearity ≥ 0.99), with each point increase associated with 30% higher VA risk [HR 1.32 (1.26–1.39)], and a clear threshold effect observed around score 5 above which hazard ratios consistently exceeded 1.0 (*Figure 1B*).

**Figure 1:**
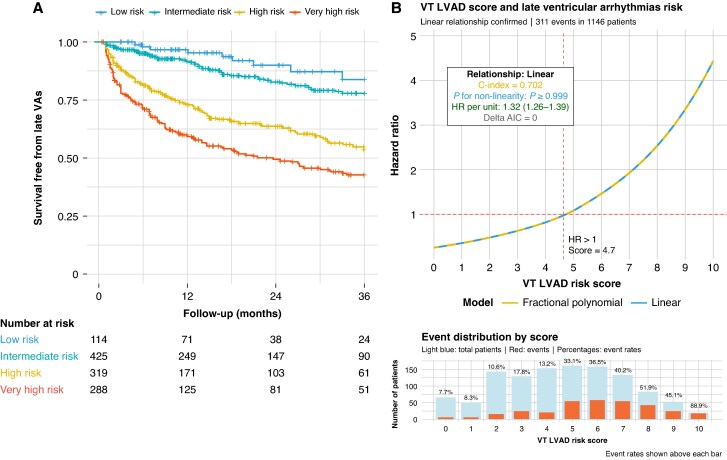
Risk stratification and prediction of late ventricular arrhythmias according to the VT-LVAD score. (*A*) Kaplan–Meier curves showing freedom from late VAs across VT-LVAD risk groups over 3-year follow-up. (*B*) Relationship between VT-LVAD score and late VA risk. AIC, Akaike information criterion; HR, hazard ratio; LVAD, left ventricular assist device; VA, ventricular arrhythmia.

To date, this represents the largest international study validating the VT-LVAD score for predicting late VAs in LVAD recipients. Our main findings are as follows: (i) the cumulative incidence of late VAs reaches 27.1%, confirming this as a common complication; (ii) the VT-LVAD score, based on six easily accessible clinical variables, demonstrated good performance in risk stratification; and (iii) each 1-point increase in the score was associated with 30% higher late VA risk, with a clear threshold effect around score 5.

These results have important clinical implications for individualizing patient management. Specifically, while the survival benefit of ICD therapy in this population remains unproven,^1,5^ ICD implantation is widespread with nearly 50% of LVAD patients having ICDs prior to device placement,^5^ making risk-based decision-making guided by the VT-LVAD score particularly relevant. Rather than a generalized approach, our findings suggest individualized decision-making based on intrinsic arrhythmic risk assessment, where ICD therapy should be strongly considered in patients classified as high or very high risk (score ≥ 5), whereas those with low scores may not benefit from ICD therapy since, beyond the lack of demonstrated survival benefit, ICD in LVAD recipients is associated with high risks of potential complications including inappropriate shocks, electromagnetic interference, infections, and pocket haematomas.^1,5,6^ Beyond ICDs, the VT-LVAD score may also help identify candidates for prophylactic catheter ablation at the time of LVAD implantation, a strategy currently being evaluated in the PIVATAL trial.^7^ Future prospective studies should determine whether risk-stratified management strategies guided by this score can improve patient outcomes and optimize therapeutic decisions.

Our study has several limitations. First, the retrospective design may have introduced selection bias, and patients without ICDs may have experienced undetected self-terminating VAs, though this reflects real-world practice. Moreover, we lacked detailed data on antiarrhythmic therapies and catheter ablation during follow-up.

In conclusion, this large international multicentre study validates the VT-LVAD score as a reliable tool for predicting late VA risk in LVAD recipients. The score demonstrated good discrimination and can be used to individualize risk stratification in clinical practice. Future studies should evaluate whether risk-stratified management strategies guided by this score can optimize therapeutic decisions, particularly regarding ICD implantation and prophylactic catheter ablation.

## Data Availability

The datasets used and/or analysed during the current study are available from the corresponding author on reasonable request.
